# A Systematic Review of Network Studies Based on Administrative Health Data

**DOI:** 10.3390/ijerph17072568

**Published:** 2020-04-09

**Authors:** Shakir Karim, Shahadat Uddin, Tasadduq Imam, Mohammad Ali Moni

**Affiliations:** 1 Complex Systems Research Group, Faculty of Engineering, The University of Sydney, Darlington, NSW 2008, Australia; 2 School of Business and Law, CQUniversity, L4, 120 Spencer Street, Melbourne, VIC 3000, Australia; t.imam@cqu.edu.au; 3 WHO Collaborating Centre on eHealth, School of Public Health and Community Medicine, Faculty of Medicine, The University of New South wales, Sydney, NSW 2052, Australia; m.moni@unsw.edu.au

**Keywords:** administrative health data, network study, network method

## Abstract

Effective and efficient delivery of healthcare services requires comprehensive collaboration and coordination between healthcare entities and their complex inter-reliant activities. This inter-relation and coordination lead to different networks among diverse healthcare stakeholders. It is important to understand the varied dynamics of these networks to measure the efficiency of healthcare delivery services. To date, however, a work that systematically reviews these networks outlined in different studies is missing. This article provides a comprehensive summary of studies that have focused on networks and administrative health data. By summarizing different aspects including research objectives, key research questions, adopted methods, strengths and weaknesses, this research provides insights into the inherently complex and interlinked networks present in healthcare services. The outcome of this research is important to healthcare management and may guide further research in this area.

## 1. Introduction

Administrative health data are an important and the largest source of data collected from a large number of healthcare services provided by different healthcare stakeholders to patients [1,2]. These data are created when a healthcare customer connects with healthcare elements; for example, visiting a doctor, having medical diagnoses performed, being conceded into a medical clinic, or purchasing medicines from a pharmacy. The term ‘administrative health data’ also refers to “administrative data", "claim data", "electronic hospital record", “digital health data”, “digitized health data”, "electronic medical data" and "e-medical data". These data are used for patients’ care, diagnostic information, health treatments, or crosswise over various medicinal service offices (e.g., emergency clinics, aged care centers and nursing homes).

Recently, administrative health data have been used extensively in the healthcare community for research investigation and clinical decision making; for example, for disease risk prediction, analysis and restorative treatment [2]. Customarily, clinical choices have relied upon doctors’ estimation, capacity, experience and different clinical and diagnostic test outcomes. This practice culture, however, may prompt unsolicited favoritisms, faults, high admission cost and low service quality rendered to patients [2]. The combination of medical decision-making support with administrative claim data can significantly lessen medical errors and undesirable varieties of patient experiences, which could potentially increase patient safety [3]. Computer-supported information systems can play a significant role in this regard and have already seen increased application in healthcare data management [4]. A central aspect of such computerized healthcare information system is administrative health or claim data.

Administrative health data are used in a wider range of healthcare research as opposed to only for database record management; for example, medicinal services investigation [5], performance measurement of hospital care networks [6], identifying comparative cost of effective care [7], developing models for forecasting disease risk [8,9], observation of chronic illness [10] and monitoring sickness reappearance and medication results [11]. Additionally, these data are a significant resource for the examination and observation of chronic sicknesses [10].

Due to security and privacy issues, clinical datasets have very limited access permission; whereas, administrative datasets are available principally to multiple healthcare stakeholders. Access to these data occur through varied network architectures by varied healthcare entities. Further to patients’ health records, medicinal history and referral information [4], authoritative healthcare data captures these interactions including patient-doctor interactions, patient-nurse interactions, patient-pharmacist interactions, pharmacist-doctor interactions, and patient-medical center communications,

There is, however, a lack of a study that assesses these networks in an integrated fashion. This study is a wide-ranging systematic review of networks dependent on administrative health data. By assessing existing works, different types of networks have been identified in this study. The aims, methods and measures utilized in various articles are also noted and related objectives have been summarized. Moreover, the strengths and limitations of the different methods are examined. The outcomes of this study will assist different healthcare stakeholders (e.g., researchers, academics, patients, health care and insurance providers and Government) to quickly conceptualize developments that have occurred concerning networks using administrative health data and allow informed decision making. Further, since administrative datasets have wide application for research purposes, the outcomes will also guide future investigations.

## 2. Methodology

The search strategy of this study considered two keywords. They are: “administrative data” and “network”. Since the first keyword (i.e., “administrative data”) may appear in different forms in the present literature, we utilized different synonyms for this keyword. These synonyms are “administrative claim data”, “administrative health data”, “electronic health data” and “claim data”. For the second keyword (i.e., “network”), we did not consider any synonyms. This is because all synonyms that were used for this keyword in the existing literature (i.e., “network measure”, “network study”, “network review”, “network analysis” and “network comprehensive analysis”) contain the word “network”. This led to the development of the following search term for this study: *("administrative data" OR “administrative claim data” OR “administrative health data” OR “electronic health data” OR "claim data") AND “network".*

**Figure 1:**
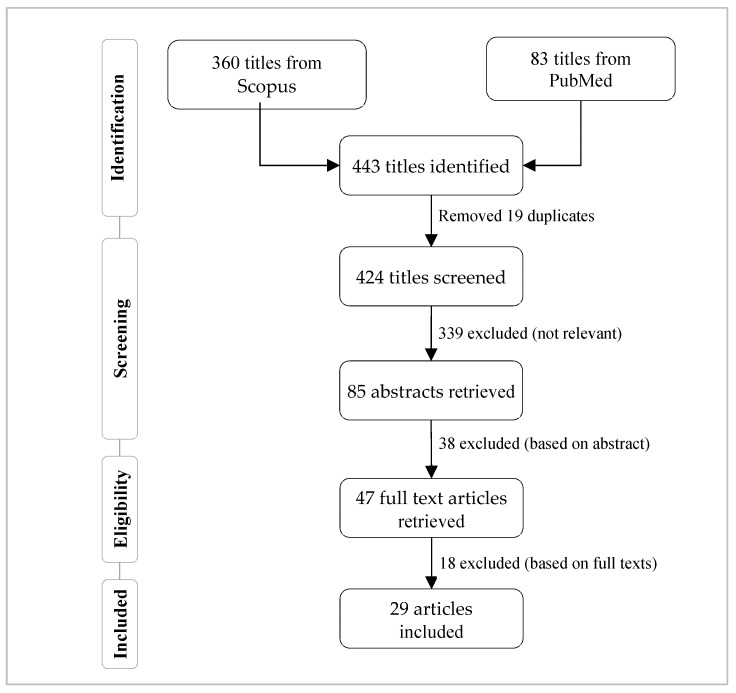
Flowchart for the selection of articles that were reviewed in this study.

**Figure 2:**
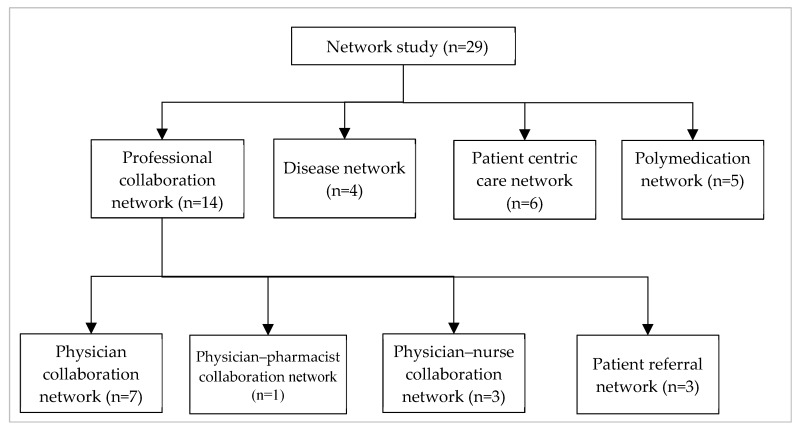
The list and number of various types of network studies (based on administrative health data) that were reviewed in this study.

The above complex search term was scanned for articles in PubMed and Scopus databases. The metadata (i.e., title, abstract and keywords) of each scholarly article were considered during this search. We found 587 and 83 articles from PubMed and Scopus, respectively. We followed the steps described by DuGoff et al. [12] in selecting the articles that were considered to review in this study. After removing 19 duplicate titles, we screened the remaining 424 articles. We considered only those articles that are written in English and published in peer-reviewed journals or conferences. After this screening, 339 articles were excluded as they are not relevant to this study. In most cases, for conducting a network study, these 339 articles considered health data other than the administrative one. Subsequently, we manually reviewed the abstract first, and then the full text of the underlying articles to make the final selection of the 29 articles reviewed in this study. The complete flowchart of the study selection process followed in this study is depicted in Figure 1. These 29 articles were then split into different categories according to the type of healthcare stakeholders involved in the underlying studies. This categorization is presented in Figure 2.

## 3. Emergence of Network in Healthcare

**Figure 3:**
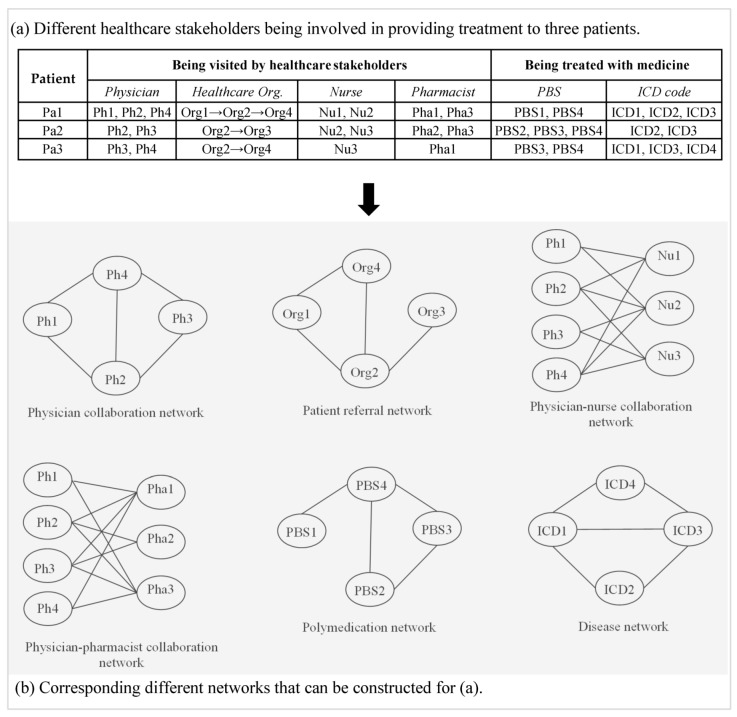
Construction of different networks based on an abstract administrative health dataset for three patients: (**a**) treatment information by different stakeholders; and (**b**) corresponding different healthcare stakeholder networks. Here *Pa* stands for patient, *Ph* for physician, *Org* for organisation that provides healthcare to patients, *Nu* for nurse, *Pha* for pharmacist, *PBS* for pharmaceutical benefits scheme and *ICD* for international classification of diseases.

Networks can emerge in healthcare in different ways. A physician collaboration network (PCN), for example, can emerge among physicians when providing healthcare services to common patients. Such networks can also emerge when physicians visit common hospitalized patients [13]. In the current literature, such a network is also known as the patient-sharing network among physicians. In a similar way, other networks can emerge in healthcare among varied stakeholders involved. Based on an abstract dataset about the treatment information of three patients, Figure 3 provides an illustration of the construction of different networks emerged among various healthcare stakeholders while providing treatment to patients. As in Figure 3a, physicians *Ph1*, *Ph2* and *Ph4* visited patient *Pa1*, patient *Pa2* is seen by physicians *Ph2* and *Ph3*, patient *Pa3* is visited by physicians *Ph3* and *Ph4*. The top-left network shows the corresponding PCN. In this PCN, there are network connections between *Ph1* and *Ph2*, *Ph1* and *Ph4*, *Ph2* and *Ph3*, *Ph2* and *Ph4*, and *Ph3* and *Ph4* since they visit one or more common patients. Other networks constructed in Figure 3 follow a similar network construction logic for the respective healthcare setting. The following subsections provide details concerning these networks.

### 3.1. Professional Collaboration Network

Collaboration is a significant part of group healthcare [14] and is complex with numerous traits including the sharing of arrangements, decision making, tackling of issues, defining of objectives and acceptance of obligations [15,16]. Collaborative networks among people and groups are profoundly esteemed in associations in light of the fact that consolidating multi-dimensional endeavours and assorted expertise produce benefits more prominent than those accomplished through individual effort [17,18]. In a professional collaboration network, nodes represent healthcare service providers (e.g., physician, nurse, pharmacist and healthcare providing organisation), links between nodes indicates that the healthcare service providers have provided healthcare services to one or more common patients and the weights of those links represent the number of common patients. Different professional collaboration networks are found in the healthcare literature that used administrative claim data. They are briefly described in the following.

#### 3.1.1. Physician Collaboration Network

It is a basic practice in the healthcare business that when doctors visit patients daily, they provide directions concerning treatments depending on the ailment and the past and recorded therapeutic history. Past advice from physicians is also taken into consideration during any shadow visits by other doctors to that patient. This comprehensive healthcare practice empowers academics to plot and prototype a physician collaboration network (PCN). There are many studies in the present healthcare literature that explore the impact of PCN structure on various healthcare outcomes [1,19,20]. For example, Uddin et al. [21] projected a classical model that utilizes specific engagement such as physician–physician relationships to acquire knowledge about actual collaboration and coordination in healthcare.

#### 3.1.2. Physician–Pharmacist Collaboration Network

The collaboration between physicians and pharmacists has been explored to check whether such collaborations can make any difference to the concerned healthcare outcome(s). Chobanian et al. [22] conducted a study to assess whether, or not, a synergistic model between a doctor and a group of pharmacists in network-based workplaces could improve blood pressure (BP) control. In their study, clinical pharmacists made some medical treatments and drug suggestions to doctors, and medical attendants performed regular BP checking. They found that the synergistic model between a doctor and pharmacists in network-based workplaces can improve BP control.

#### 3.1.3. Physician–Nurse Collaboration Network

A physician–nurse collaboration network evolves when a group of physicians and nurses provide healthcare services to one or more patients. A related research, undertaken by Knaus and his group, recognizes a notable connection between the level of physician–nurse joint effort and patient mortality in intensive care units [23].

#### 3.1.4. Patient Referral Network

A patient referral network is the connections established among several healthcare service providers through referrals of patients by doctors. A patient referral network is generally a directed graph since ‘who is referring to whom’ is known. In a patient referral network, nodes are healthcare service providers and an edge (or link) between a pair of nodes is directed from the ‘visiting healthcare provider’ to the ‘referred healthcare provider’ [24,25].

### 3.2. Disease Network

Disease networks represent the development of different diseases, usually captured by the international classification of diseases (ICD) codes [26], within individual patients in a given period. In such networks, a node represents an individual disease or a comorbid condition, a link between a pair of nodes indicates that one or more patients developed the disease conditions represented by those nodes and the weight of a link represents the number of patients being treated for the diseases that are represented by the end nodes of that link. Khan et al. [27] developed a baseline disease network for type 2 diabetic patients by using their comorbid conditions captured by the Elixhauser index [28]. By applying graph theory and complex network methods, they used this baseline network for the predictive risk analysis of type 2 diabetes. A baseline disease network represents the succession of comorbidities sustained by patients derived from the restorative service histories.

### 3.3. Patient-Centric Care Collaboration Network

Different service providing units provide healthcare services to patients during their hospitalization periods. These include doctor–specialist units, pathology and diagnosis centers and health services units of the underlying healthcare service provider. In addition, triage doctors monitor patients depending on the emergency concerning their extreme urgencies [6]. A network between patient-centric care entities therefore emerges at the time of patients’ admissions and subsequent hospitalization periods [6]. For a given patient, a patient-centric care network and its different member nodes (actors) indicate the healthcare services she received during her hospitalization period from different hospital units. It further renders the level of engagement of these hospital units in providing healthcare services to that patient.

### 3.4. Polymedication Network

A polymedication network is produced from a regimen which comprises of a treatment plan and medical actions [29]. Such networks can be captured from the drug intake history of patients. Typically, different coding methods are followed to store patients’ drug intake records in different countries. The pharmaceutical benefits scheme (PBS), for example, is followed in Australia. The corresponding polymedication network of the abstract data (second last column) of Figure 3a is depicted in the lower middle figure of Figure 3b. A node of this network indicates the PBS code of the underlying medicine. A link between two nodes indicates that at least one patient was prescribed with the two medicines represented by those nodes. There is a potential relationship among patients’ age, medical and pharmaceutical expenses, and polymedication networks can assist pharmacovigilance and distinguishing adverse medication responses [29].

## 4. Results and Discussions

Since this study provides a literature review of network studies that used administrative health data, we first describe different network measures and methods that were used in our reviewed articles. This will ease to follow our findings in this study. Accordingly, Table 1 briefly outlines different network measures and methods under seven broad categories. These categories are node-level measure, network-level measure, edge-level measure, exponential random graph model, cohesive subgroup analysis, community analysis, and dyad and triad census analysis.

As illustrated in Figure 2, only one article based on the physician–pharmacist collaboration network met the search criteria of this study. However, there are many studies in the literature that analysed physician–pharmacist collaboration networks to address various research questions. Studies exploring such networks mostly used randomized control trials or survey methods to collect the corresponding research data [52,53]. Overall, for conducting this literature review, we did not find many articles that met our search criteria. Administrative health data were merely available to researchers globally even a couple of years before due to privacy reasons. This unavailability of administrative health data for research purposes may be a possible reason for this shortage of such articles. Many administrative data are now increasingly becoming available to researchers. The findings from this study implies that there is a scope for further research in this field, especially for less explored networks like the physician–pharmacist network.

**Figure 4:**
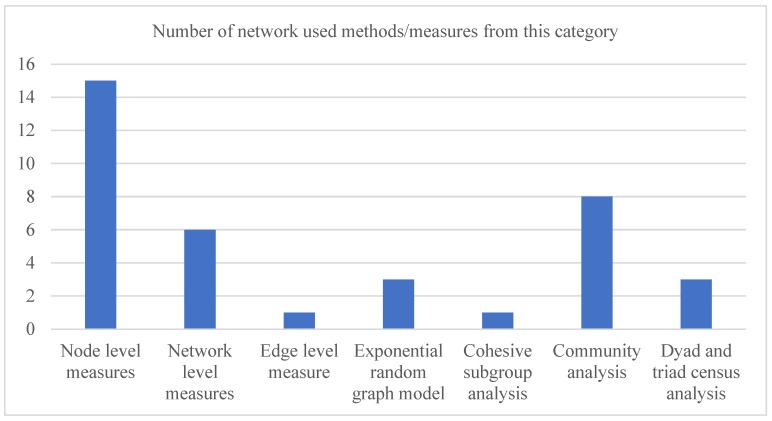
Frequency of different network measures and methods that were used by the 29 articles considered in this study. Some of these articles employed network measures and methods from more than one category.

As notable from Table 2, most studies have focused on the network structure of different professional collaborations and their impact on various performance measures (e.g., cost, length of stay and quality of care). Table 3 shows the key findings of each article reviewed in this study. It also illustrates the network measures or methods employed in each reviewed article. In the majority of the cases, the structure of the underlying network is correlated with the corresponding study goals. In few cases, socio-demographic attributes (e.g., patient age, patient sex and hospital geography) have been found to impact the development of the underlying healthcare professional networks. Based on the categorization of major network methods and measures as in Table 1, Figure 4 illustrates the frequency of different network measures and methods that were used by the 29 articles considered in this study. Finally, Table 4 outlines the strength and weaknesses of seven different type of networks identified in this study.

Except the articles that belong to the last two categories of Table 3 (i.e., Disease network and Polymedication network), all articles reviewed in this study analyzed different networks among various healthcare stakeholders (individuals or organizations) to understand how network structure affects the perceived level of different treatment outcomes. For example, all articles reviewed under the PCN category analyzed physician collaborations using diverse network measures. The ultimate goal of these studies was to figure out the PCN structure that is more conducive to better treatment outcomes. On the other side, the articles belonging to the ‘Disease network’ category attempted to map the co-appearance of different diagnostic codes in order to get a better understanding of the progression of a single disease or comorbidity of multiple diseases. All articles under the ‘Polymedication network’ category explored the complex relationships among various drugs.

Although all articles considered in this study conducted network analysis utilizing administrative health data and different network measures, a close examination of Table 3 shows a comprehensive relationship between the network utilized in the various investigations and their key findings. Community analysis and exponential random graph model were mostly utilized in investigations that intended to explore diverse coordination and joint efforts for different health conditions.

It is evident, from Figure 4, that the ‘*node level measures*’ has been used more often (15 times) in the literature. Commonly used measures of this category were degree centrality and betweenness centrality. The second most employed category of network measures and methods is the ‘*community analysis*’. There are eight reviewed articles that used this method. The ‘*network level measures*’ stands as the third most used category. The measures of this category have appeared in six reviewed articles. The common measures of this category included network density and network centralization. Notably, only limited focus has been made in literature on *‘edge level measure’* and *‘cohesive subgroup analysis’*—again, an indication that there are opportunities to undertake further research in these areas.

## 5. Conclusions

This study provides a complete review of network studies based on only administrative claim data. We utilized the search technique explained in the method section to extract the 29 articles considered in this study. All reviewed articles of this study utilized administrative health data to conduct a network study. The findings of this study can be used by healthcare policymakers in developing future research strategies. They can also be used by prospective future researchers for a summarized comprehension of the current research on network studies that use administrative health data.

Similar to other review studies, this study may miss articles that utilized administrative health data for a network study. This could be a possible limitation of this study. However, this limitation will not block the essential point of this study in giving an image of administrative health data utilization for network studies. This review examined various network studies that used administrative claim data. A comprehensive understanding of different networks can provide insights that are important for improving healthcare systems. The capability of administrative health data in terms of their usefulness in conducting network study is also effectively demonstrated in this study, which could be a great extent for healthcare research.

## Figures and Tables

**Table 1:** Explanation of major network methods and measures across different aspects.

Aspects	Methods and Measures	Definition
Node level measure	Degree, closeness, betweenness, eigenvector and other similar measures	Degree centrality: It depicts the number of ties a node (or actor) has with other nodes in a network. It can be of two types (in-degree and out-degree) in a directed network [30]. Closeness centrality: For a node, it represents the extent it is close to the remaining nodes in a network [30]. Betweenness centrality: It represents the extent an actor is in a favoured position in terms of falling on the shortest paths between other actor pairs in the network [30]. Eigenvector: It measures the influence of a node in a network and can distinguish the degree centrality from cases where nodes having a wide range of degree values are connected [30].
Network level measure	Network centralization, density, network diameter and other similar measures	Network centralization: The centralization of a network indicates how central its most central node is compared with how central other nodes within that network are [30]. Network density: It represents the ratio between the number of existing links in a network and the total number of possible links that can be presented among all network actors [30]. Network diameter: It represents the size of the largest path in a network [30].
Edge level measure	Tie strength	Tie strength: It represents the strength of relation between a pair of actors in a network [30] and can be quantified from their duration of relation and the reciprocal services (that specify their tie) they have in common [31].
Exponential random graph model	This model and its different variants	Exponential random graph model: It is a probabilistic model that can identify the building blocks of a given network with respect to different micro-level network substructures (e.g., dyad, triangle and 3-star) [32].
Cohesive subgroup analysis	Clique, clan, n-clique, n-clan and other similar measures	Clique: A clique is a group of actors or nodes in a network that are directly connected with each other [30]. n-clique: An n-clique is also a clique where all member nodes are reachable to each other through at most (n-1) intermediate member nodes [30]. n-clan: An n-clan is also a clique where all member nodes are reachable to each other through at most (n-1) intermediate nodes [30]. The intermediate nodes may or may not be a member of the clique.
Community analysis	Community detection	Community detection: It helps to identify a group of nodes in a network that are densely connected among themselves but sparsely connected with other nodes of that network [30].
Dyad and triad census analysis	Dyad and triad census	A dyad is a subgraph comprising two nodes or actors, while a triad is a subgraph consisting of three actors. Both dyads and triads can be formed with or without any links between their member actors [30]. Various dyadic and triadic structures (known as dyad and triad census) are used to explore the building block of networks.

**Table 2:** List of studies that focus on networks using administrative health data. This study considers only those study that used administrative health data to conduct a network study in a healthcare context. Network studies based on other health data (e.g., survey data) have been excluded.

Network Type	Research Question(s)	Reference
Physician collaboration network (PCN)	- How does the microscale level structure among physicians affect hospitalization cost and emergency clinic readmission rate?	Uddin et al. [33]
	- What network attributes of PCN affect hospitalization expense and readmission rate? - How does the PCN structure ease the effective delivery of healthcare services to patients?	Uddin et al. [19]
	- How can a comprehensive connection being built among physicians when sharing patients correspond with their professional relationships?	Barnett et al. [34]
	- What correspondence and connection exist between different healthcare collaboration and coordination networks?	Uddin et al. [1]
	- How do the attributes of patient-sharing physician collaboration networks improve health results?	Uddin et al. [20]
	- Do the expert networks among physicians shift crosswise over geographic areas? - How do physician professional networks impact elements that are related to their associations?	Landon et al. [13]
	- Can coordination between physicians reduce expenses of care and probability of hospitalization?	Pollack et al. [35]
Patient-centric care coordination network	- What effects do the progressions in structural places of actors have in a short interim and aggregated network?	Uddin et al. [36]
	- How do different attributes make a significant impact on hospitalization cost and hospital length of stay? - How can a network capture coordination between patient-centric care services during patient hospitalization period?	Uddin [37]
	- How does a social network-based research framework enhance collaborative performance under various healthcare settings?	Uddin and Hossain [38]
	- Does patient-centred care network impact hospitalization cost?	Uddin and Hossain [6]
	- How do patient-physician tie quality and patient sociodemographic factors influence the social structure of tasks and conveyance of financially savvy healthcare services?	Abbasi et al. [39]
	- What characteristics of a patient-driven network produce effective clinical outcomes?	Uddin et al. [21]
Physician –nurse collaboration network	- How can physician–nurse collaboration scale be used to quantify the impression of joint practice among medical attendants and doctors?	Caricati et al. [40]
	- How does the physician–nurse collaborative relationship affect patients’ mortality and length of stay?	Tschannen and Kalisch [41]
	- How do physician and nurse form collaboration network and what improvements can be achieved in terms of the nature of medical services from such networks?	Yao et al. [42]
Physician-pharmacist collaboration network	- How can collaboration between doctors and pharmacists improve management of chronic conditions?	DeMik et al. [43]
Patient referral network	- How do small-scale and full-scale design of patient referrals under the US patient referral networks motivate future healthcare developments? - What is the motivation of future healthcare developments?	An et al. [24]
	- Can network analysis find appropriate referral networks for healthcare organizations?	Vukmir et al. [25]
	- What impacts various patient referral designs have on the potential spread of emergency clinics between various classes of medical organizations?	Donker et al. [44]
Disease network	- How network-based approach and clinical regulatory information can assist in building up a portrayal of chronic disease movement?	Khan et al. [45]
	- How accurately a disease prediction framework can predict the risk of chronic diseases?	Khan et al. [46]
	- How can comorbidity patterns enhance the understanding of different risk factors for chronic diseases?	Khan et al. [27]
	- How does the comorbidity of multiple chronic ailments lead to the progression of cardiovascular conditions?	Hossain and Uddin [47]
Polymedication network	- Is managerial information useful to recognize drug regimens from discrete data of medication dispenses?	Khan et al. [29]
	- How does a polymedication network help healthcare system administrators to assemble the maps of diagnostics and recommend drugs with regards to constant ailments and polypharmacy?	Zamora et al. [48]
	- How can pharmacological mechanisms be investigated using a poly-dimensional network?	Liu et al. [49]
	- What association between the attributes of patients and health service providers lead to the utilization of paediatric psychotropic polypharmacy?	Medhekar et al. [50]
	- How should specialists review available data to understand patients and the relevant therapeutic practice?	Franchini et al. [51]

**Table 3:** Characteristics (i.e., network methods followed and key findings) of the included network studies.

Network Type	Reference	Network Methods/Measures Used	Key Findings
Physician collaboration network (PCN)	Uddin et al. [33]	Exponential random graph model	- The network structure of PCN has an impact on different patient outcomes (e.g., healthcare expense and hospitalization readmission rate).
	Uddin et al. [19]	Network centralization	- The degree centrality and network density of PCNs impact hospitalization cost and readmission rate.
	Barnett et al. [34]	Community detection	- A positive correlation has been found between the strength of professional relationship among physicians and the number of shared patients. - This correlation is stronger for primary care physicians compared to medical or surgical specialists.
	Uddin et al. [1]	Exponential random graph model	- Exponential random graph model can explore the collaborative endeavours of different healthcare stakeholders.
	Uddin et al. [20]	Triad census, Clique and Clan	- The triad census and subgroup statistics of PCNs can predict hospitalization cost, hospital length of stay and readmission rate.
	Landon et al. [13]	Network centrality	- The collaboration pattern among physicians varies across geographic areas. Physicians who have characteristics in common tend to share patients among themselves.
Patient-centric care coordination network	Uddin et al. [36]	Closeness centrality	- A model is introduced for investigating the impact of network position of patients, physicians and clinic actors on healthcare outcomes in a patient-driven care network.
	Uddin [37]	Community detection	- The number of physicians engaged in delivering healthcare services to patients has a positive association with hospitalisation cost. Patient age, gender and comorbidity score moderated this association. - The community structure and network density of physicians’ joint efforts can explain the varied healthcare outcomes across different hospitals.
	Uddin and Hossain [38]	Dyad and Network centrality	- Social network attributes of network centrality, connectedness and tie strength are correlated with the coordination performance of patient-centric care networks. This relation is moderated by patient age, patient sex and hospital type.
	Uddin and Hossain [6]	Connectedness, Degree centrality and Tie strength	- Network positions of patients, physicians and hospital actors have an impact on hospitalization expense.
	Abbasi et al. [39]	Network centrality	- Network centrality measures of a patient-centric network can explore the operation and delivery of cost-effective healthcare services.
	Uddin et al. [21]	Network centrality and Exponential random graph model	- By extracting and analysing networks among various healthcare stakeholders, this study proposed policies for cost-effective healthcare environments.
Physician–nurse collaboration network	Caricati et al. [40]	Community detection	- Physicians valued collaborative practices more than nurses. Also, collaborative practices were acknowledged to a lesser extent in contexts with higher standardization and automation (e.g., in critical care units).
	Tschannen and Kalisch [41]	Network centrality	- Physician–nurse collaboration is positively linked with the actual length of stay. - A care from a physician–nurse collaboration may lead to a longer length of stay but very effective for the treatment of different complications.
	Yao et al. [42]	Network centrality	- This study associated network measures with specific healthcare team behaviours. It also identified interventions for improving the design of healthcare teams and the training of healthcare professionals towards an enhanced quality of patient care.
Physician–pharmacist collaboration network	DeMik et al. [43]	Community detection	- A correlation is present between existing clinical pharmacy services and provider attitudes and beliefs in regard to implementing a novel pharmaceutical intervention.
Patient referral network	An et al. [24]	Network centrality and Triad census	- Through a better understanding of network features, patient referral networks can provide insights for developing the US healthcare system.
	Vukmir et al. [25]	Community detection	- Patients often show low compliance with follow-up recommendations, even being directed by the emergency department patient referral system.
	Donker et al. [44]	Degree centrality and Community detection	- This study predicts that (a) it is very likely that hospital-acquired infections will rapidly spread from one hospital to other hospital(s); and (b) For such spreads, hospitals that are being referred must be ready for a rapid response.
Disease network	Khan et al. [45]	Network centrality	- The understanding of the comorbid conditions that lead to diabetes can effectively be used for developing better health policy and resource management.
	Khan et al. [46]	Network centrality	- Chronic disease network is a novel approach to deal with the danger of type 2 diabetes. Such networks offer a methodology that can be utilized by private healthcare organizations and Governments for an improved and viable use of administrative health data.
	Khan et al. [27]	Network centrality	- The mapping of diagnostic codes and their co-appearances helps in constructing comorbidity networks and, thereby, aids in understanding the progression pattern of chronic diseases at a population level. - Targeted preventive health management programs can be planned and designed to reduce hospital admissions and associated cost.
	Hossain and Uddin [47]	Network centrality	- Occurrence of blood and kidney diseases is related to cardiovascular diseases for type 2 diabetic patients.
Polymedication network	Khan et al. [29]	Network centrality	- A complex relationship among various drugs can capture the multimorbidity nature of different targeted treatments. - The polymedication regimens that are expanded over a long time can be used for the treatment of chronic conditions (e.g., diabetes and asthma).
	Zamora et al. [48]	Betweenness centrality and Community detection	- Chronic, polymedicated patients require special attention because of the prevalence of high treatment cost and the associated risks. - There are identifiable patterns between joint diagnostics and associated drugs.
	Liu et al. [49]	Network centrality	- Pharmacological mechanism of baicalein is influenced by varied medical factors.
	Medhekar et al. [50]	Community detection	- “Pediatric psychotropic polypharmacy” is necessary and its prescription by providers is well justified.
	Franchini et al. [51]	Network density	- Network analysis can assist identifying complex associations between large scale patient information that can otherwise remain undetected. - The study identified crucial factors that need to be considered for the development of clinical guidelines.

**Table 4:** The strength and weakness of different types of network.

Network Type	Strength	Weakness
Physician collaboration network	- It can capture longitudinal collaborative network structures developed among physicians while providing treatment to patients. - Able to quantify the networked role of each physician, which will eventually ease in developing better healthcare policy.	- It cannot capture the information regarding whether physicians discuss the concerned patient in person, or they develop a common understanding of the patient’s medical condition through medical prescriptions, clinical reports and diagnostic outcomes.
Patient-centric care coordination network	- It can capture the network connectivity among different healthcare agencies (e.g., hospital and rehabilitation) that are engaged in providing treatment to patients. - It can identify the number of health services provided by each healthcare agency engaged in providing treatments.	- Since this is an ego-centric network (patients are at the center of the network), some network analysis approaches (e.g., betweenness centrality) cannot be applied in exploring such networks.
Physician–nurse collaboration network	- It can provide special healthcare to high risk patients, which is essential for eliminating errors and promoting high quality. - It can leverage existing knowledge sources to assess contrasts and similarities between physicians and nurses.	- It cannot explain the different dimensions of collaborative practice that evolve between nurses and physicians. - It is unable to recognize the real degree of a joint effort.
Physician–pharmacist collaboration network	- It can strengthen healthcare results and improve the comprehension of physician–pharmacist relationship in a primary care setting. - It eases collaboration among professionals working in different drug stores.	- This network does not incorporate many facilities with geographic, racial and financial assorted varieties, which is important for the execution of group-based management for different diseases.
Patient referral network	- It can capture the entire journey of a patient with geographical proximities during receiving treatments across different healthcare service providers. - It can help in developing healthcare policies (e.g., developing new healthcare facilities in a new area), which will eventually reduce patients’ traveling distance in accessing different healthcare services.	- It cannot explain why patients travel variable distances for accessing healthcare services. - It could be the case that a patient traveled a long distance to access a healthcare service from a provider although the patient can access the same service from another provider by traveling a much shorter distance.
Disease network	- It helps in understanding the progression different disease conditions and their comorbidities. - It eases the prediction of disease risk without any clinical or pathological tests. - It empowers healthcare providers in developing preventive health management projects to diminish clinical and other related expenses.	- It cannot conceptualize other significant covariates (e.g., smoking status, alcohol consumption and functional impartment) that might be related to exacerbation risk. - It underestimates hospital-acquired complications in exploring disease progressions. - It does not allow the accurate classification of medication errors that may cause clinical complications.
Polymedication network	- It can contribute in deciding the gathering of health variables and diagnostics that are generally pertinent in the population. - It can characterize new pointers of population health that mirror the unpredictability of current situations. - It can potentially identify the adverse effects of various drugs.	- It cannot combine the medication dispensed events enlisted in the pharmaceutical benefits scheme data and recognize drug regimens.
